# Effect of fluoride varnishes on oral bacteria of preschool children with cavitated and non-cavitated carious lesions: randomized clinical trial

**DOI:** 10.1038/s41598-023-45636-9

**Published:** 2023-10-29

**Authors:** Sheetal Manchanda, Divesh Sardana, Simin Peng, Edward C. M. Lo, Cynthia K. Y. Yiu

**Affiliations:** 1 Faculty of Dentistry, The University of Hong Kong, Hong Kong, S.A.R, People’s Republic of China; 2 University of Oklahoma College of Dentistry, Oklahoma, USA; 3 Faculty of Dentistry, The University of Hong Kong, Hong Kong S.A.R, People’s Republic of China; 4 Faculty of Dentistry, University of Hong Kong, Hong Kong S.A.R, People’s Republic of China

**Keywords:** Applied microbiology, Bacteria

## Abstract

We compare the effect of calcium and phosphate-containing sodium fluoride (NaF) varnishes to conventional NaF varnish on *S. mutans* and *L. fermentum* counts. 3–4 years old children were grouped according to their caries status (n = 45 each): caries-free, with non-cavitated and with cavitated lesions. Each group was randomly subdivided (n = 15 each) into: Group 1- 5% NaF, Group 2- 5% NaF with tricalcium phosphate, Group 3- 5% NaF with casein phosphopeptide- amorphous calcium phosphate. Biofilm and saliva were collected to quantify microorganisms at baseline (T1) and 24-months (T2). Differences between groups were compared using Kruskal–Wallis test, followed by Dunn-Bonferroni post-test, at 0.0167 α-level. Significant difference was found for percentage of children with detectable biofilm *L. fermentum* (*p* = 0.013) at T1 and salivary *S. mutans* (*p* = 0.011) at T2. Percentage of children increased from T1 to T2 in Group 2 with salivary *S. mutans* (*p* = 0.007), salivary *L. fermentum* (*p* = 0.035), and biofilm *L. fermentum* (*p* = 0.019) and in Group 3 with salivary *L. fermentum* (*p* = 0.035). Bacterial change was not significant in both samples of intervention groups, except increase in salivary *S. mutans* (*p* = 0.038) in Group 3. Both calcium- and phosphate-containing NaF varnishes demonstrated similar antibacterial effect on *S. mutans* and *L. fermentum* compared to conventional NaF varnish.

## Introduction

Early childhood caries (ECC) is regarded as a multifactorial dietary-microbial disease with bacteria in the dental biofilm as an important etiologic factor^1^. Despite various preventive measures, ECC remains a health issue worldwide, with millions of children with untreated caries in their primary teeth^2^. Clinically, the caries severity stages are defined according to the break in the continuity of enamel surface as non-cavitated or cavitated lesions^3^ Earlier studies^4,5^ have focused on demineralization at the cavitation level, with only a few longitudinal studies^6^ evaluating the progression of non-cavitated lesions to cavitation over time. A recent review highlighted the importance of identifying and preventing non-cavitated lesions from progressing to cavitation by non-operative interventions^3^.

An interaction between sugars and biofilm microorganisms adhered to the tooth surface^7^ produces acids that reduce the pH, leading to demineralization of the tooth surface by favouring loss of calcium and phosphate ions from the enamel^8^. On the contrary, saliva plays an essential role in promoting enamel remineralization due to the presence of mineral ions and antimicrobial proteins^9^ and prevents demineralization by neutralizing biofilm pH due to its buffering capacity and flushing of food debris^7^. Among the various factors involved in caries, microorganisms are essential for its occurrence and progression^10^. *Streptococcus mutans* (*S. mutans*)^11^ and *Lactobacillus fermentum* (*L. fermentum*)^12,13^ are the major pathogenic ones involved as they have been regarded as the microbial risk markers of ECC^14^.

Numerous caries-preventive measures impact the oral microflora directly or indirectly, among which fluoride is the most effective^15^ as it strengthens the crystalline structure of enamel^16^ and has antibacterial properties against certain cariogenic microorganisms^17,18^. Additionally, fluoride is known to inhibit bacterial colonization in plaque and various bacterial enzymes like enolase, acid phosphatase, pyrophosphatase, peroxidase and catalase^19^. Fluoride varnish has the advantage of providing a longer contact time for action on the tooth surface^20^, with evidence^21,22^ supporting its effectiveness in caries reduction; thus, it is recommended for preventing caries in both primary and permanent dentition^23^. Conventional varnish containing 5% sodium fluoride (NaF) has been found to retard demineralization and promote enamel remineralization by uptake of calcium and phosphate ions from saliva^24^. However, limited bioavailability of these ions in the conventional NaF varnish, led to the development of novel NaF varnishes that provides a constant supply of ions for enhancing the subsurface remineralization^25^. Among these is a NaF varnish containing amorphous calcium phosphate with casein phosphopeptide (CPP-ACP), which has a significant remineralizing effect due to its ability to maintain a supersaturated form by acting as a reservoir of bio-available calcium and phosphate^26–28^. Another recently introduced varnish containing tricalcium phosphate (TCP) in addition to 5% NaF has been reported to be effective in preventing ECC among preschool children^29^. However, these novel agents' in vivo antibacterial effect on *S. mutans* and *L. fermentum* has not been investigated much.

Previous study has reported the fluoride varnishes effect on microbial count, attributing the decline in microbial levels to the presence of additional calcium ions in the varnishes that contributes to a high alkaline pH^4^. Another in vitro study^30^ conferred this decrease in *S. mutans* count to the pellicle modification caused by CPP, inhibiting its initial adhesion on the tooth surface and its combination with fluoride enhances this anti-adhesion effect. Earlier studies mainly reported varnish effect on bacterial count by culturing methods using colony-forming unit (CFU) counts^31,32^ or by commercially available strip tests^4,5^. However, no published study has used quantitative Real-time Polymerase Chain Reaction (qRT-PCR), which is an accurate and specific method for detecting targeted bacteria due to the use of species-specific primers^33^. Therefore, the present study aimed to compare the effect of NaF varnishes containing calcium and phosphate to that of conventional NaF varnish on *S. mutans* and *L. fermentum* count in saliva and biofilm of preschool children who were caries-free, with non-cavitated and cavitated lesions. The null hypothesis stated that there is no difference in the effect of fluoride varnishes containing calcium and phosphate on *S. mutans* and *L. fermentum* count compared to the conventional fluoride varnish.

## Methods

### Sample size calculation

Sample size estimation was performed using G*Power 3.1 software (Heinrich Heine University Düsseldorf, Germany) based on an earlier study^4^, which was a randomized clinical trial comparing the antibacterial activity of three different fluoride varnishes on salivary bacterial levels in children with severe ECC. The effect size for the 3 groups compared at 3 months interval for *S. mutans* and *L. fermentum* counts were 0.43 and 0.48, respectively. Therefore, based on the average effect size of 0.31 from the earlier study^4^, a sample size of 35 in each group was required to achieve a power of 80% at a 5% statistical significance level. Accounting for the drop-out, 45 children were needed in each group (135 in total).

### Study design and population

Children in this study were recruited from a larger randomized controlled clinical trial conducted in the kindergartens of Hong Kong, with an optimal fluoride drinking water concentration of 0.5 ppm. The kindergartens were selected by purposive sampling from a list of non-profit-making kindergartens obtained from the Hong Kong Education Bureau. To ensure generalizability of the findings and for the control of confounders, stratified random sampling generated a pool of 135 participants from the clinical trial, with 45 in each group. The trial obtained ethical approval from Institutional Review Board of the University of Hong Kong (reference number: UW 18–054) and was conducted in accordance with the best clinical and research practices and in accord with the declaration of Helsinki (1964). The protocol was registered at www.clinicaltrials.gov (registration number: NCT04274569; Date: 18/02/2020). Children’s parents were explained about the study, and written informed consent was obtained before the sample collection or clinical examination. The study is being reported following the 2010 CONSORT statement and checklist (www.consort-statement.org/).

Children were 3–4 years of age with no prior history of any severe medical condition, special health care needs or long-term medication. Children were excluded if they had antibiotic intake within the past month, primary teeth enamel hypoplasia, professional fluoride treatment in the past six months, hypersensitivity to any constituents in the study varnishes, or uncooperative behavior during examination or sample collection.

### Clinical examination

Children were examined in their kindergarten in a supine position using a disposable mouth mirror attached to a handle with a light-emitting diode (MirrorLite; Kudos International Holdings Limited, Hong Kong) and a 0.5 mm ball-ended probe (405/WHO probe; Otto Leibinger GmBH, Germany). A calibrated and trained dentist, unaware of the group assignment, performed caries diagnosis using International Caries Detection and Assessment System II (ICDAS II)^34^ criteria with few modifications. Since the kindergarten settings limited the availability of air drying of teeth, ICDAS code 1 was not used. The non-cavitated lesion was recorded by ICDAS code 2, indicating a distinct visual change in enamel, while ICDAS codes 3 to 6 were used to record the status of a cavitated lesion. In addition, codes 7 and 8 were used for filled teeth with or without caries, respectively, and code 9 for teeth missing due to caries. Primary teeth unerupted or missed for reasons other than caries were recorded as code 99. In addition, 10% randomly selected children were re-examined on the same day at baseline and 24-month follow-up to assess the intra-examiner reproducibility by Cohen’s Kappa.

### Interventions

Forty-five children were randomly selected in each of the following 3 groups according to their dental status: no clinical signs of caries (ICDAS code 0 on all teeth), with only non-cavitated carious lesions (ICDAS code 2 on one or more teeth but not with ICDAS code 3 or above in any teeth) and with cavitated carious lesions with or without non-cavitated lesions (ICDAS code 3 to 6 on one or more teeth). Each group was further subdivided randomly into 3 groups with 15 children each to receive one of the 3 study fluoride varnishes: Group 1 (positive control)—5% NaF (Duraphat Varnish, Colgate-Palmolive Ltd, (UK) Ltd., Guildford, Surrey, UK), Group 2 (test)—5% NaF with TCP (Clinpro White Varnish; 3 M ESPE, St Paul, MN, USA), and Group 3 (test)—5% NaF with CPP-ACP (MI Varnish; GC Corporation, Itabashi-Ku, Tokyo, Japan). The detailed composition of the varnishes used is presented in Supplementary Table 1. A computer-generated random number concealed in an opaque, sealed and labelled envelope that remained unopened until the start of the intervention (provided by a statistician not involved in the intervention or examinations) was used to randomly allocate children into one of the 3 intervention groups by an assistant not involved in the examination or intervention. Children, parents and examiner were unaware of the treatment allocation throughout the study. A trained clinician, different from the one who performed the examination, was the intervention provider who applied the assigned study varnish (0.25 mL per child) using disposable micro brushes on all erupted primary teeth after removing visible plaque and drying teeth with gauze. Due to the nature of intervention with different colours and smell of the varnishes, the intervention provider could not be blinded. Kindergarten teachers were instructed not to serve any drinks or food to the children within the next 2–4 h and to inform their parents about refraining from brushing their children’s teeth and eating any rough, hard food for next 24 h. Each child received a toothpaste containing 600 ppm fluoride (Colgate Minions Toothpaste) and a manual toothbrush (Colgate—Smiles Minions Kids Toothbrush) in each follow-up visit.

Children were planned to receive 8 applications of the assigned fluoride varnish quarterly over 24 months. However, COVID-19 pandemic led to the inability of the research team to comply with the intended fluoride varnish application protocol. As a result, total number of applications received by the children ranged from 3 to 6 due to refusal of parents for varnish applications and closure of kindergartens.

### Sample collection

Salivary and biofilm *S. mutans* and *L. fermentum* levels were determined at baseline and 24-month follow-up. Supragingival biofilm was collected from the bucco-gingival surfaces of all erupted primary teeth using sterile cotton buds, which were then placed in a microcentrifuge tube containing 1 mL Phosphate buffered saline (PBS) (pH 7.4). In addition, 1 mL unstimulated whole saliva was collected on the same occasion after biofilm collection by asking the child to spit directly into a sterile falcon plastic tube. Sample collection was performed at least an hour after the food intake and was then immediately cold-transported to the laboratory for storage at −70 °C until the DNA extraction procedure.

### Microbiological procedure

Samples were centrifuged for 10 min at 9800 rpm, following which the supernatant was discarded. The bacterial pellet was then suspended in 20 mM Tris–HCl, pH: 8.0; 2 mM EDTA; 1.2% Triton (Triton X-100) and cells lysed with 20 mg/ml lysozyme, followed by overnight incubation in a water bath at 37 °C. Finally, manufacturer’s instructions were followed for DNA extraction procedure using QIAamp Mini DNA kit (Qiagen, Hilden, Germany), and the extracted DNA was then stored at −20 °C until further use.

ABI StepOnePlus Real-Time PCR system (Applied Biosystems, USA) was used to perform the reaction in Microamp fast optical 96-well reaction plates (Applied Biosystems, Foster City, CA, USA) covered with optical adhesive film (Applied Biosystems). The cycle conditions were: 50 °C /2 min; 95 °C /10 min; 50 cycles of 95 °C /15 s and 58 °C /1 min. The oligonucleotide primers used in the study are listed in Supplementary Table 2. The TaqMan probes (Applied Biosystems, USA) used for *S. mutans*, *L. fermentum,* and Universal were 5′-FAM-TGGAAATGACGGTCGCCGTTATGAA-TAMRA-3′, 5′-FAMATGGCTTCTCGCTATC-TAMRA-3′ and 5′-FAMCGTATTACCGCGGCTGCTGGCAC-TAMRA-3′ respectively. For each 20 μl final reaction volume, the mixture was composed of 5 μl of bacterial DNA, 2 μl of deionized water, 10 μl TaqMan Universal PCR Mix (Applied Biosystems, USA), and 1 μl of forward (F) and reverse (R) primers and TaqMan Probe each. The reference bacterial DNA was serially diluted, ranging from 1 × 10^4^ to 1 × 10^9^ CFU/ml for both *S. mutans* and *L. fermentum* and was used as standards and positive controls for quantifying bacteria. All reactions were performed in duplicates with negative controls without a template.

After the amplification of the extracted DNA samples, the obtained Ct values were plotted against the standard curves, and the absolute number of *S. mutans* and *L. fermentum* in the samples were determined. Samples were considered negative for the respective bacterial species when the Ct values were below the level of detection in the DNA standard curves.

### Outcome measure

The outcome measure was the change in the salivary and biofilm *S. mutans* and *L. fermentum* levels after the application of different fluoride varnishes over 24 months follow-up.

### Statistical analysis

Data tabulation and analysis were performed on MS Office Excel 2016 (Microsoft Office Professional Plus 2016, Microsoft, USA) and SPSS v. 24 (IBM SPSS. Statistics Inc, USA), respectively. The Kolmogorov–Smirnov test was used to assess data normality. Kruskal–Wallis test, followed by post hoc pairwise comparisons using the Dunn-Bonferroni approach, was used to compare the differences between the groups. Difference in the dichotomous variable between two related groups was evaluated using McNemar test, while paired-sample t-test was used for continuous dependent variable. For the categorical data, Pearson’s Chi-square test was used to assess the differences among the groups. The statistical significance level for all tests was 0.05, except for the adjustment at *p* = 0.0167 for the post-test pairwise comparison. Cohen’s kappa coefficient was used to determine the intra-examiner reproducibility at the tooth surface level.

## Results

### Description of the study population

Flow diagram of the study design conducted in March-June 2019 is presented in Supplementary Fig. 1, including 135 children, with 45 in each of the 3 groups according to their dental status and 15 in each of the 3 fluoride varnish intervention subgroups. Baseline characteristics of the recruited participants with microbial quantification in oral samples are presented in a previously published article^35^. The intra-examiner reproducibility for the clinical examination was 0.92 and 0.87 at baseline and 24-month follow-up, respectively, as calculated by Cohen’s Kappa coefficient, indicating an excellent agreement.

### Identification of microorganisms

Table 1 presents the percentage of children across the 3 intervention groups with detectable levels of both bacteria in biofilm and saliva at baseline (T1) and 24-month follow-up (T2). Statistically significant difference was found between groups for the percentage of children with detectable biofilm *L. fermentum* at T1 (*p* = 0.013) and salivary *S. mutans* at T2 (*p* = 0.011). At T1, the percentage of children identified with *S. mutans* in saliva and biofilm and for salivary *L. fermentum* did not significantly differ among the groups (*p* > 0.05). Similarly, at T2, the percentage of children with detectable salivary and biofilm *L. fermentum* and biofilm *S. mutans* were not statistically different among the groups. However, in Group 2 (NaF + TCP), there was an increase in the percentage of children with detectable salivary *S. mutans* (*p* = 0.007) and *L. fermentum* (*p* = 0.035) and biofilm *L. fermentum* (*p* = 0.019) between T1 and T2. Also, the percentage of children identified with *L. fermentum* (*p* = 0.035) in saliva increased between T1 and T2 in Group 3 (NaF + CPP-ACP).

**Table 1:** Frequency of identification [n (%)] of *S. mutans* and *L. fermentum* in biofilm and saliva samples using qRT-PCR at baseline (T1) and 24- month follow-up (T2) in the study groups [^*^McNemar test, ^**^Pearson Chi-square test; SM-*Streptococcus mutans*, LF- *Lactobacillus fermentum*].

Intervention groups (n)	Saliva SM (T1)	Saliva SM (T2)	*p*-value*	Saliva LF (T1)	Saliva LF (T2)	*p*-value*	Biofilm SM (T1)	Biofilm SM (T2)	*p*-value*	Biofilm LF (T1)	Biofilm LF (T2)	*p*-value*
Duraphat (45)	23 (51.1%)	27 (60.0%)	0.454	26 (57.7%)	32 (71.1%)	0.210	18 (40.0%)	18 (40.0%)	1.000	25 (55.5%)	26 (57.7%)	1.000
Clinpro White (45)	26 (57.7%)	37 (82.2%)	**0.007**	24 (53.3%)	33 (73.3%)	**0.035**	27 (60.0%)	25 (55.5%)	0.791	15 (33.3%)	26 (57.7%)	**0.019**
MI Varnish (45)	18 (40.0%)	24 (53.3%)	0.109	23 (51.1%)	34 (75.5%)	**0.035**	20 (44.4%)	21 (46.6%)	1.000	12 (26.6%)	20 (44.4%)	0.096
p-value**	0.234	**0.011**		0.812	0.893		0.137	0.333		**0.013**	0.343	

Significant values are in bold.

### Quantification of the change in salivary and biofilm microorganisms

*S. mutans* and *L. fermentum* levels among the 3 intervention groups at T1 and T2 are shown in Table 2. No significant difference was found between T1 and T2 for both *S. mutans* and *L. fermentum* levels in biofilm samples in all the study groups (*p* > 0.05). Similarly, in salivary samples, no significant difference was observed between T1 and T2 for both *S. mutans* and *L. fermentum* levels in all 3 groups except in Group 3 (NaF + CPP-ACP), where the salivary *S. mutans* level was higher at T2 than at T1 (*p* = 0.038).

**Table 2:** Salivary and biofilm bacterial values (in CFU/ml) in three study groups at baseline (T1) and 24-month final follow-up (T2) [Paired sample T-test; SM-*Streptococcus mutans*, LF- *Lactobacillus fermentum,* SD- Standard deviation].

Intervention groups (n)		Salivary parameters						Biofilm parameters					
		SM (T1)	SM (T2)	*p*-value	LF (T1)	LF (T2)	*p*-value	SM (T1)	SM (T2)	*p*-value	LF (T1)	LF (T2)	*p*-value
Duraphat (45)	Mean	1.79E + 06	7.77E + 05	0.506	6.42E + 04	1.48E + 05	0.402	1.46E + 05	1.78E + 04	0.081	9.65E + 02	3.62E + 03	0.285
	SD	9.87E + 06	3.69E + 06		3.11E + 05	5.86E + 05		4.77E + 05	5.82E + 04		3.15E + 03	1.61E + 04	
	Median	3.02E + 02	1.79E + 03		2.73E + 02	1.20E + 03		0.0	0.0		6.32E + 01	8.95E + 01	
	1^st^ Quartile	0.0	0.0		0.0	0.0		0.0	0.0		0.0	0.0	
	3^rd^ Quartile	7.62E + 04	1.53E + 05		3.46E + 03	1.28E + 04		2.85E + 04	2.22E + 03		3.76E + 02	3.23E + 02	
Clinpro White (45)	Mean	1.08E + 06	2.58E + 06	0.188	2.88E + 04	3.81E + 04	0.645	7.55E + 05	1.59E + 05	0.112	3.94E + 03	5.40E + 02	0.294
	SD	2.96E + 06	8.75E + 06		1.13E + 05	8.59E + 04		2.42E + 06	8.96E + 05		2.14E + 04	1.33E + 03	
	Median	1.40E + 03	5.31E + 04		2.03E + 02	1.58E + 03		3.79E + 02	4.85E + 02		0.0	8.08E + 01	
	1^st^ Quartile	0.0	7.21E + 02		0.0	0.0		0.0	0.0		0.0	0.0	
	3^rd^ Quartile	2.74E + 05	7.01E + 05		7.99E + 02	2.52E + 04		9.01E + 04	1.09E + 04		2.70E + 02	5.10E + 02	
MI Varnish (45)	Mean	1.33E + 06	2.30E + 06	**0.038**	2.74E + 04	2.20E + 05	0.090	6.25E + 05	2.41E + 04	0.158	3.09E + 03	3.77E + 03	0.767
	SD	4.97E + 06	6.67E + 06		1.04E + 05	7.41E + 05		2.89E + 06	9.14E + 04		1.40E + 04	1.11E + 04	
	Median	0.0	2.98E + 02		8.65E + 01	7.16E + 02		0.0	0.0		0.0	0.0	
	1^st^ Quartile	0.0	0.0		0.0	2.23E + 01		0.0	0.0		0.0	0.0	
	3^rd^ Quartile	1.11E + 04	1.18E + 06		5.81E + 02	3.44E + 04		2.60E + 04	8.42E + 03		1.23E + 02	7.33E + 02	

Significant values are in bold.

Table 3 presents the change in salivary and biofilm bacterial levels in each group from T1 to T2. No statistically significant difference was observed for the change in *S. mutans* and *L. fermentum* levels in both saliva and biofilm after the application of any of the 3 fluoride varnishes (*p* > 0.05).

**Table 3:** Change in salivary and biofilm parameters in each study group from baseline (T1) to 24-month final follow-up (T2) [Kruskal Wallis test; SM-*Streptococcus mutans*, LF- *Lactobacillus fermentum,* SD- Standard deviation].

	Intervention groups	Change from T1-T2					
		Mean	S.D	Median	1^st^ Quartile	3^rd^ Quartile	*p*-value
Saliva SM	Duraphat	− 1.02E + 06	1.01E + 07	0.0	− 7.79E + 03	1.15E + 04	0.113
	Clinpro White	1.50E + 06	7.54E + 06	9.57E + 02	− 9.87E + 02	1.52E + 05	
	MI Varnish	9.68E + 05	3.03E + 06	0.0	0.0	7.49E + 05	
Biofilm SM	Duraphat	− 1.28E + 05	4.79E + 05	0.0	− 3.35E + 03	0.0	0.362
	Clinpro White	− 5.96E + 05	2.47E + 06	− 1.77E + 02	− 3.83E + 04	0.0	
	MI Varnish	− 6.01E + 05	2.81E + 06	0.0	− 1.15E + 04	0.0	
Saliva LF	Duraphat	8.35E + 04	6.62E + 05	0.0	− 2.86E + 02	8.96E + 03	0.795
	Clinpro White	9.32E + 03	1.35E + 05	2.61E + 02	− 2.52E + 02	2.09E + 04	
	MI Varnish	1.93E + 05	7.46E + 05	1.20E + 02	− 1.06E + 02	2.14E + 04	
Biofilm LF	Duraphat	2.66E + 03	1.65E + 04	0.0	− 2.36E + 02	2.01E + 02	0.618
	Clinpro White	− 3.40E + 03	2.15E + 04	0.0	0.0	2.25E + 02	
	MI Varnish	6.74E + 02	1.52E + 04	0.0	0.0	3.21E + 02	

Table 4 presents the mean change in the salivary and biofilm bacterial levels across the three study groups according to the child’s caries status. No significant difference was observed in the mean *S. mutans* and *L. fermentum* levels in saliva and biofilm in all 3 groups. Change in *S. mutans* and *L. fermentum* levels in both saliva and plaque samples from T1 to T2 for each study group has been presented in the form of line graphs in Supplementary Figs. 2, 3, 4, 5.

**Table 4:** Change in the salivary/biofilm parameters in 3 study groups according to the caries status of the child from baseline (T1) to 24-month final follow-up (T2) [Kruskal Wallis test; SM-*Streptococcus mutans*, LF- *Lactobacillus fermentum,* SD- Standard deviation].

Caries status	Intervention group (n)		Salivary SM change	*p*-value	Salivary LF change	*p*-value	Biofilm SM change	*p*-value	Biofilm LF change	*p*-value
Caries-free	Duraphat (15)	Mean (SD)	9.40E + 04 (3.95E + 05)	0.505	6.30E + 03 (1.89E + 04)	0.304	−6.81E + 03 (1.34E + 05)		−4.70E-01 (1.18E + 02)	0.404
		Median (1st Quartile, 3^rd^ Quartile)	0.00 (0.00, 7.05E + 03)		1.62E + 02 (0.00, 2.27E + 03)		0.00 (-2.55E + 02, 0.00)	0.505	0.00 (-6.32E + 01, 3.73E + 01)	
	Clinpro White (15)	Mean (SD)	−2.96E + 04 (7.76E + 05)		4.06E + 04 (1.20E + 05)		−4.41E + 03 (1.34E + 04)		4.93E + 02 (2.19E + 03)	
		Median (1st Quartile, 3^rd^ Quartile)	1.62E + 03 (0.00, 1.09E + 05)		1.83E + 03 (0.00, 2.01E + 04)		0.00 (-2.87E + 03, 7.95E + 02)		0.00 (0.00, 7.11E + 02)	
	MI Varnish (15)	Mean (SD)	9.79E + 05 (2.17E + 06)		6.35E + 04 (2.39E + 05)		−2.09E + 04 (1.09E + 05)		3.09E + 03 (1.15E + 04)	
		Median (1st Quartile, 3^rd^ Quartile)	0.00 (0.00, 6.11E + 05)		1.19E + 02 (-7.74E + 01, 2.61E + 03)		0.00 (0.00, 3.04E + 02)		0.00 (0.00, 1.78E + 02)	
Non-cavitated	Duraphat (15)	Mean (SD)	1.56E + 06 (6.01E + 06)	0.519	8.62E + 03 (5.99E + 05)	0.700	−1.21E + 04 (6.05E + 04)	0.567	2.54E + 03 (8.33E + 03)	0.520
		Median (1st Quartile, 3^rd^ Quartile)	0.00 (0.00, 8.56E + 04)		1.40E + 02 (−2.58E + 02,2.34E + 04)		0.00 (−1.69E + 02, 3.75E + 02)		0.00 (−3.82E + 02, 2.42E + 03)	
	Clinpro White (15)	Mean (SD)	4.67E + 06 (1.24E + 07)		4.61E + 04 (8.13E + 04)		−3.37E + 05 (2.36E + 06)		5.06E + 02 (1.16E + 03)	
		Median (1st Quartile, 3^rd^ Quartile)	1.59E + 04 (0.00, 5.70E + 05)		4.04E + 02 (0.00, 9.67E + 04)		0.00 (−2.61E + 04, 0.00)		2.18E + 01 (0.00, 3.97E + 02)	
	MI Varnish (15)	Mean (SD)	3.46E + 04 (7.28E + 04)		5.01E + 04 (1.24E + 05)		−1.62E + 04 (7.08E + 04)		1.34E + 03 (2.94E + 03)	
		Median (1st Quartile, 3^rd^ Quartile)	0.00 (0.00, 5.05E + 03)		1.69E + 03 (0.00, 3.61E + 04)		0.00 (0.00, 0.00)		0.00 (0.00, 9.67E + 02)	
Cavitated	Duraphat (15)	Mean (SD)	−4.70E + 06 (1.63E + 07)	0.083	2.36E + 05 (9.91E + 05)	0.784	−3.64E + 05 (7.81E + 05)	0.530	5.44E + 03 (2.77E + 04)	0.663
		Median (1st Quartile, 3^rd^ Quartile)	−3.54E + 04 (−1.29E + 06, 6.39E + 04)		−2.81E + 02 (−6.55E + 04, 1.92E + 04)		−2.06E + 02 (−3.80E + 05, 0.00)		0.00 (−1.05E + 03, 3.55E + 02)	
	Clinpro White (15)	Mean (SD)	−1.31E + 05 (2.91E + 06)		−5.87E + 04 (1.70E + 05)		−1.45E + 06 (3.51E + 06)		−1.12E + 04 (3.67E + 04)	
		Median (1st Quartile, 3^rd^ Quartile)	0.00 (−4.70E + 05, 4.48E + 04)		−2.74E + 02 (-3.68E + 04, 0.00)		−1.17E + 04 (−1.07E + 06, −1.23E + 02)		0.00 (−7.14E + 02, 1.14E + 02)	
	MI Varnish (15)	Mean (SD)	1.89E + 06 (4.73E + 06)		4.64E + 05 (1.25E + 06)		−1.77E + 06 (4.75E + 06)		−2.41E + 03 (2.38E + 04)	
		Median (1st Quartile, 3^rd^ Quartile)	8.87E + 05 (0.00, 3.25E + 06)		0.00 (−4.17E + 04, 9.95E + 04)		−2.27E + 04 (−1.68E + 06, 0.00)		0.00 (−1.48E + 03, 3.29E + 02)	

### Analysis between bacterial levels and the number of fluoride applications

**Figure 1:**
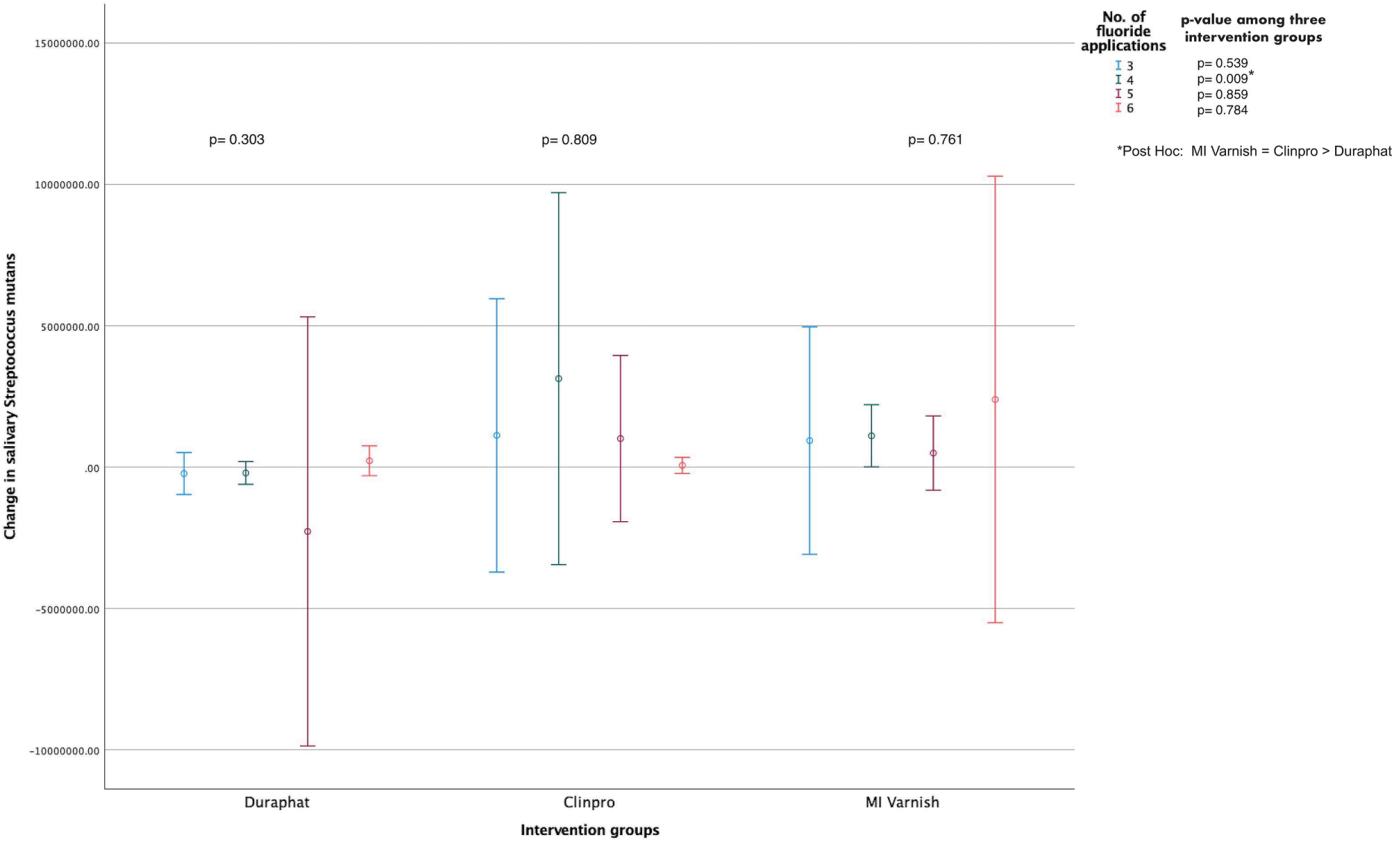
Mean change (95% CI) in the salivary *S. mutans* levels (CFUs/mL) within and between the study groups stratified according to the total number of fluoride varnish applications [N = 135 (45 in each group); Independent-Samples Kruskal Wallis Test].

**Figure 2:**
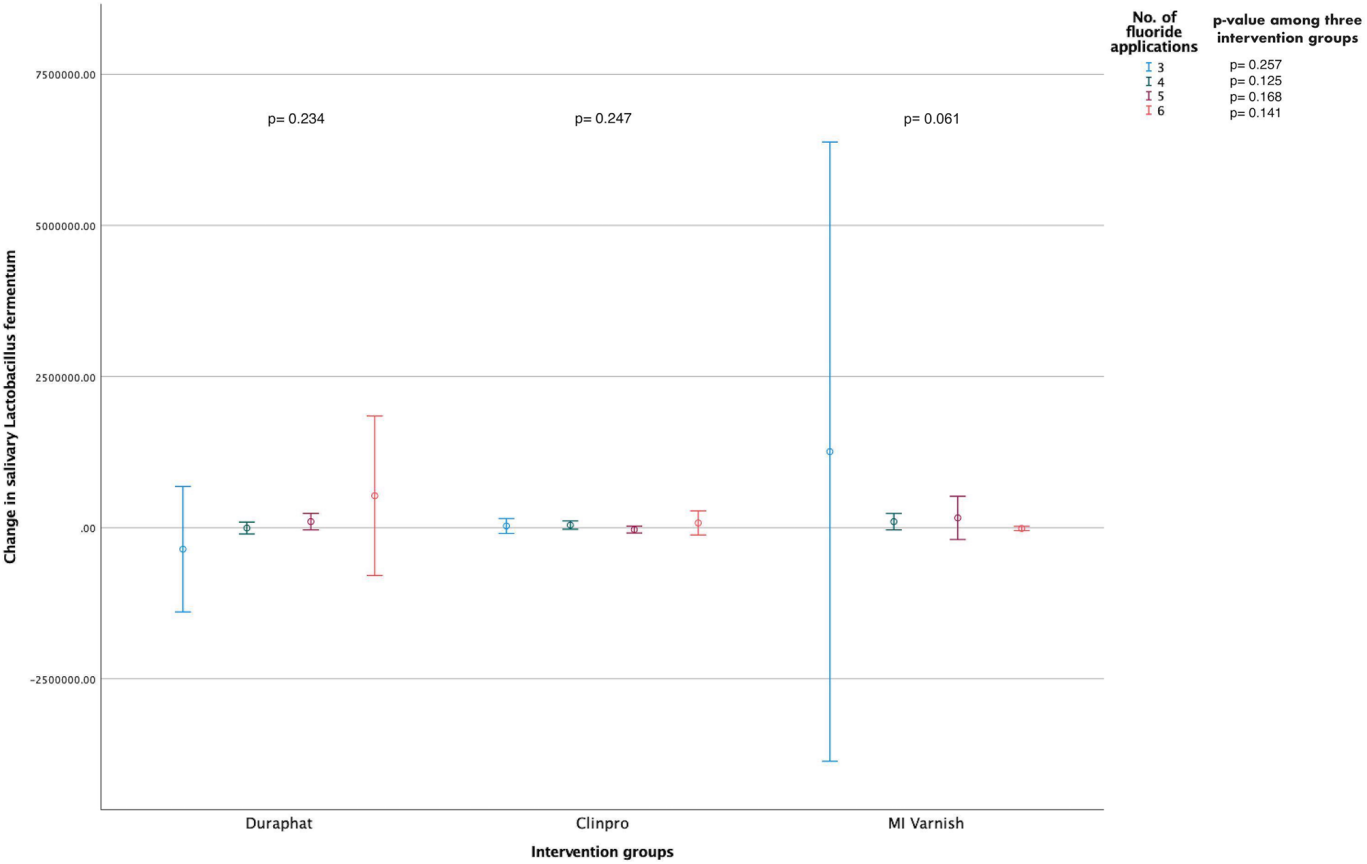
Mean change (95% CI) in the salivary *L. fermentum* levels (CFUs/mL) within and between the study groups stratified according to the total number of fluoride varnish applications [N = 135 (45 in each group); Independent-Samples Kruskal Wallis Test].

**Figure 3:**
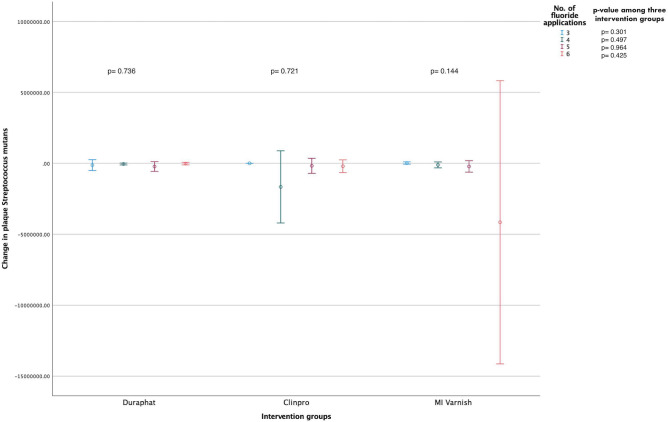
Mean change (95% CI) in the biofilm *S. mutans* levels (CFUs/mL) within and between the study groups stratified according to the total number of fluoride varnish applications [N = 135 (45 in each group); Independent-Samples Kruskal Wallis Test].

**Figure 4:**
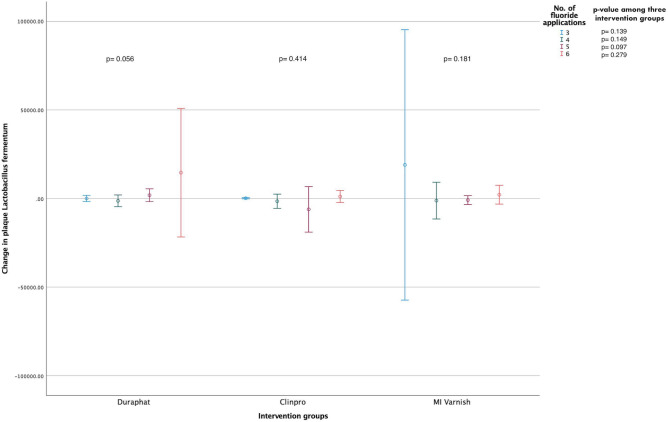
Mean change (95% CI) in the biofilm *L. fermentum* levels (CFUs/mL) within and between the study groups stratified according to the total number of fluoride varnish applications [N = 135 (45 in each group); Independent-Samples Kruskal Wallis Test].

Due to COVID-19 pandemic during the study, none of the children could receive all the planned fluoride varnish applications at 3-month intervals. Figures 1, 2, 3 and 4 presents the sensitivity analysis done to evaluate the change in the salivary and biofilm *S. mutans* and *L. fermentum* levels within and between each group, stratified according to the total number of applications received by the child. No significant difference was found for the change in bacterial levels in both samples within each intervention group. Moreover, the changes in the salivary and biofilm bacterial levels were not statistically significant for the different number of varnish applications among each group, except for the change in salivary *S. mutans,* which significantly differ among the intervention groups for children receiving four varnish applications (*p* = 0.009). In the multiple comparisons, it was found that the increase in *S. mutans* counts in saliva was least in the control group compared to the two test groups. Supplementary Figs. 6, 7, 8 and 9 represent the box plots for the difference in the mean change in the bacterial levels across the intervention groups according to the different number of fluoride applications.

The study found no adverse effects associated with the application of fluoride varnishes used in this clinical trial.

## Discussion

The study found that the two calcium and phosphate-containing NaF varnishes have a similar effect on *S. mutans* and *L. fermentum* counts in both saliva and biofilm samples as the conventional NaF varnish. Therefore, the null hypothesis stating that there is no difference in the effect of two calcium and phosphate-based fluoride varnishes compared to the conventional fluoride varnish on bacterial counts could not be rejected.

Previous studies^4,36^ demonstrating the effect of fluoride varnishes on bacterial counts have compared the microbiota of caries-free children and with cavitated lesions but missed those with non-cavitated lesions. Classifying caries lesions according to its severity can help better understand the microbiota associated with the initiation and progression of caries^37^. Thus, the current study analysed samples collected from children who were caries-free, with non-cavitated and cavitated carious lesions and evaluated the effect of the fluoride varnishes on microbial count. To the best of our knowledge, the present study is the first randomized clinical trial evaluating the efficacy of varnishes containing NaF with additional TCP/ CPP-ACP on the bacterial count in children with different cavitation levels using qRT-PCR. Although the current study did not find any significant difference in the bacterial counts after applying both the test fluoride varnishes compared to the conventional NaF varnish, the results add significantly to the literature and the understanding of the effect of fluoride and novel agents in the prevention of ECC.

Another similar study^38^ conducted on 7–8 years old children also could not demonstrate a significant decrease in the biofilm *S. mutans* level after applying conventional NaF varnish. However, a randomized clinical trial^39^ conducted on 6–12 years old children found that fluoride varnish with additional CPP-ACP significantly reduces salivary *S. mutans* when applied every week for four consecutive weeks. These varying results may be attributed to the shorter follow-up compared to our study, difference in the frequency of varnish applications and the inclusion of different age group children. Moreover, a review^40^ found that CPP-ACP has a long-term remineralizing effect on non-cavitated lesions but fails to show any significant advantage of its addition to the fluoride-containing products. Similarly, a randomized trial^4^ compared the antibacterial activity of 5% NaF varnish with other varnishes containing 5% NaF and additional calcium and phosphate (TCP and CPP-ACP) among 3–5-year-old children by evaluating the change in *S. mutans* and *L. fermentum* counts using CRT bacteria kits. The study found that fluoride varnish with TCP had a significantly higher bacterial count reduction than other varnishes and attributed their finding to the presence of calcium ions making the pH alkaline. Different microbiological methodologies used for the bacterial assessment in children with severe ECC and the intensive regimen strategy followed with frequent varnish applications in the previous studies could have led to the differences in the results from the findings of the current study. Moreover, recently conducted reviews^41^ have reported low evidence to support the advantage of adding calcium and phosphate-based supplements to fluoride versus fluoride alone in reducing the salivary *S. mutans* count.

Unlike earlier studies assessing the bacterial levels using CFU counts via stereomicroscope/colony counter^31,32^ or CRT Bacteria^4^ or Dentocult SM strip^5^ the current study has used qRT-PCR to quantify cariogenic microorganisms, which is a key strength of the study. After collecting the baseline data, parents were informed about their children's oral hygiene and caries treatment needs. Unfortunately, the COVID-19 pandemic emerged shortly thereafter, lasting nearly two years, and the information on the dental visits was not collected. Nevertheless, for the 24-month follow-up, the additions of new fillings to the baseline were compared and found no significant difference among the three groups. The information on the clinical effects of the fluoride varnishes have been described elsewhere in detail in another manuscript, allowing better understanding of the efficacy of the preventive intervention provided during the observation period.

The study had a few limitations, like parent’s refusal of varnish application due to the fear of COVID-19 transmission and closure of kindergartens, resulting in the inability to comply with the intended number and frequency of varnish applications, which could have caused the variability in the microbial assessment outcome and comparison among groups. However, sensitivity analysis was performed and found no significant effect of the different number of fluoride varnish applications received by the children on the microbial change over the follow-up period. Whereas the effect of the time gap between varnish applications could not be explored, which might have impacted the influence of fluoride varnish applications on the bacterial counts and needs to be considered while interpreting the results of the study. In addition, the effect of fluoride on bacterial count may be underestimated based on the study findings, as the current study did not evaluate the immediate effect of fluoride on microbial level, as fluoride has shown to have an initial antibacterial effect on cariogenic microorganisms while lacking the sustainability effect. Also, our study had a longer follow-up than most of the earlier studies, which raises an important concern of fluoride resistance to *S. mutans* due to prolonged use of fluoride^42^. Another limitation of the study is that the participating children received the 600 ppm fluoride toothpaste, which is lower than the 1000 ppm recommended by the AAPD Guideline for caries prevention in high caries risk children. However, the 600 ppm toothpaste is considered a standard toothpaste for children in the Hong Kong SAR; while the 1000 ppm toothpaste is rarely available in the market. Hence, we acknowledge that the use of different fluoride concentrations of toothpaste could impact the generalizability of the study's findings.

Within the limitations of this 24-month randomized clinical trial, the following conclusions can be made:

1. The two calcium and phosphate-containing NaF varnishes have a similar antibacterial effect on *S. mutans* and *L. fermentum* counts in both salivary and biofilm samples compared to the conventional NaF varnish when applied to 3–4 years old preschool children.
2. The current study findings raise doubt for the need for newer fluoride varnishes with additives and recommend that more well-designed clinical trials be conducted with a head-to-head comparison of different varnishes for their antimicrobial effect.

### Supplementary Information

Supplementary Information.

## Data Availability

The data supporting this study's findings are not publicly available but can be made available on reasonable request from the first author (Sheetal Manchanda).

## Abbreviations

qRT-PCR: quantitative Real-Time Polymerase Chain Reaction
*S*. *mutans*: *Streptococcus mutans*
*L. **fermentum*: *Lactobacillus fermentum*
ECC: Early Childhood Caries
ICDAS II: International Caries Detection and Assessment System II
VPI: Visible Plaque Index
AAPD: American Academy of Pediatric Dentistry
MID: Minimal Intervention Dentistry
dmfs: decayed missing filled surfaces
