# Supplementary material for: Effect of fluoride varnishes on oral bacteria of preschool children with cavitated and non-cavitated carious lesions: randomized clinical trial
Source: Sci Rep. 2023 Oct 29;13:18543. doi: 10.1038/s41598-023-45636-9 (PMC10613626; doi:10.1038/s41598-023-45636-9)
Supplement: Supplementary file 1 — Supplementary Information. [file 41598_2023_45636_MOESM1_ESM.docx]

**Supplementary information**

**Supplementary Table 1:** Detailed composition of the varnishes used in the study

| **Product** | **Manufacturer** | **Composition** |
| --- | --- | --- |
| **Duraphat Varnish** | Colgate-Palmolive, NSW, Australia | Sodium fluoride, ethanol, colophony, white wax, shellac, saccharin, other ingredients |
| **Clinpro White Varnish** | 3M ESPE, MN, USA | Pentaerythritol glycerol ester of colophony resin, n-hexane, ethanol, sodium fluoride, silica, thickener, food-grade flavour, modified tricalcium phosphate |
| **MI Varnish Varnish** | GC, Tokyo, Japan | Polyvinyl acetate, hydrogenated rosin, ethanol, sodium fluoride, CPP-ACP, silicon dioxide |

**Supplementary Table 2:** DNA primers used in the qRT-PCR analysis

| **Primer name** | **Primer sequence** | **Amplicon size (bp)** | **Source or reference** |
| --- | --- | --- | --- |
| *Streptococcus mutans* | SMUT-F: 5′-GCCTACAGCTCAGAGATGCTATTCT-3′  SMUT-R: 5′-GCCATACACCACTCATGAATTGA-3′ | 114 | [1] |
| *Lactobacillus fermentum* | LACTFER-F: 5′-TTCGCATGAACAACGCTTAAA-3′  LACTFER-R: 5′-CCGCAGGTCCATCCAGAA-3′ | 59 | [2] |
| *Universal* | UNI-F: 5′- CGCTAGTAATCGTGGATCAGAATG -3′  UNI-R: 5′- TGTGACGGGCGGTGTGTA -3′ | 69 | [1] |

**References:**

1. Yoshida, A., Suzuki, N., Nakano, Y., Oho, T., Kawada, M. & Koga, T. Development of a 5' fluorogenic nuclease-based real-time PCR assay for quantitative detection of Actinobacillus actinomycetemcomitans and Porphyromonas gingivalis. *J. Clin. Microbiol.* **41**, 863-866 (2003).
2. Dickson, E.M., Riggio, M.P. & Macpherson, L. A novel species-specific PCR assay for identifying Lactobacillus fermentum. *J. Med. Microbiol*. **54**, 299-303 (2005).

**Supplementary Figure 1:** Flow diagram of the study design


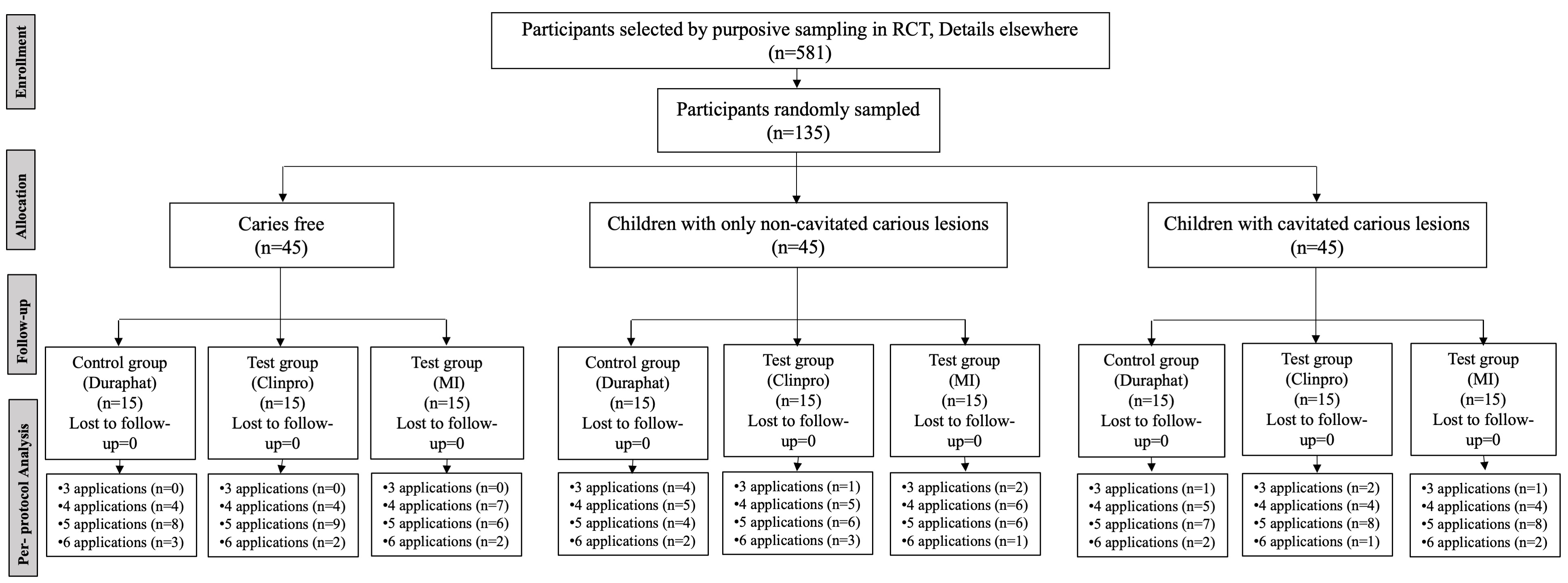


**Supplementary Figure 2: Mean change in salivary *S. mutans* across intervention groups from baseline (T1) to 24-month final follow-up (T2)**


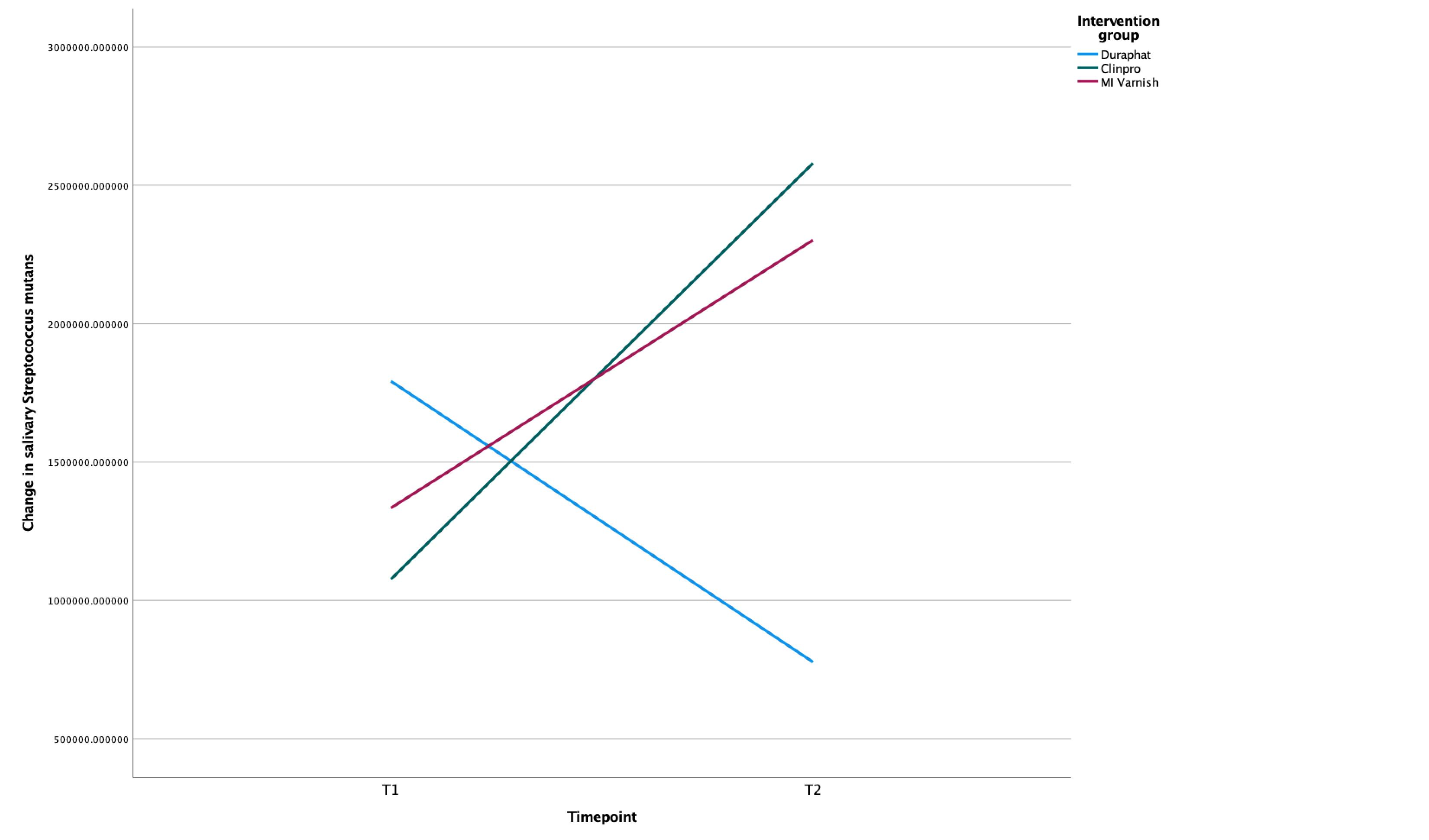


**Supplementary Figure 3: Mean change in plaque *S. mutans* across intervention groups from baseline (T1) to 24-month final follow-up (T2)**

**
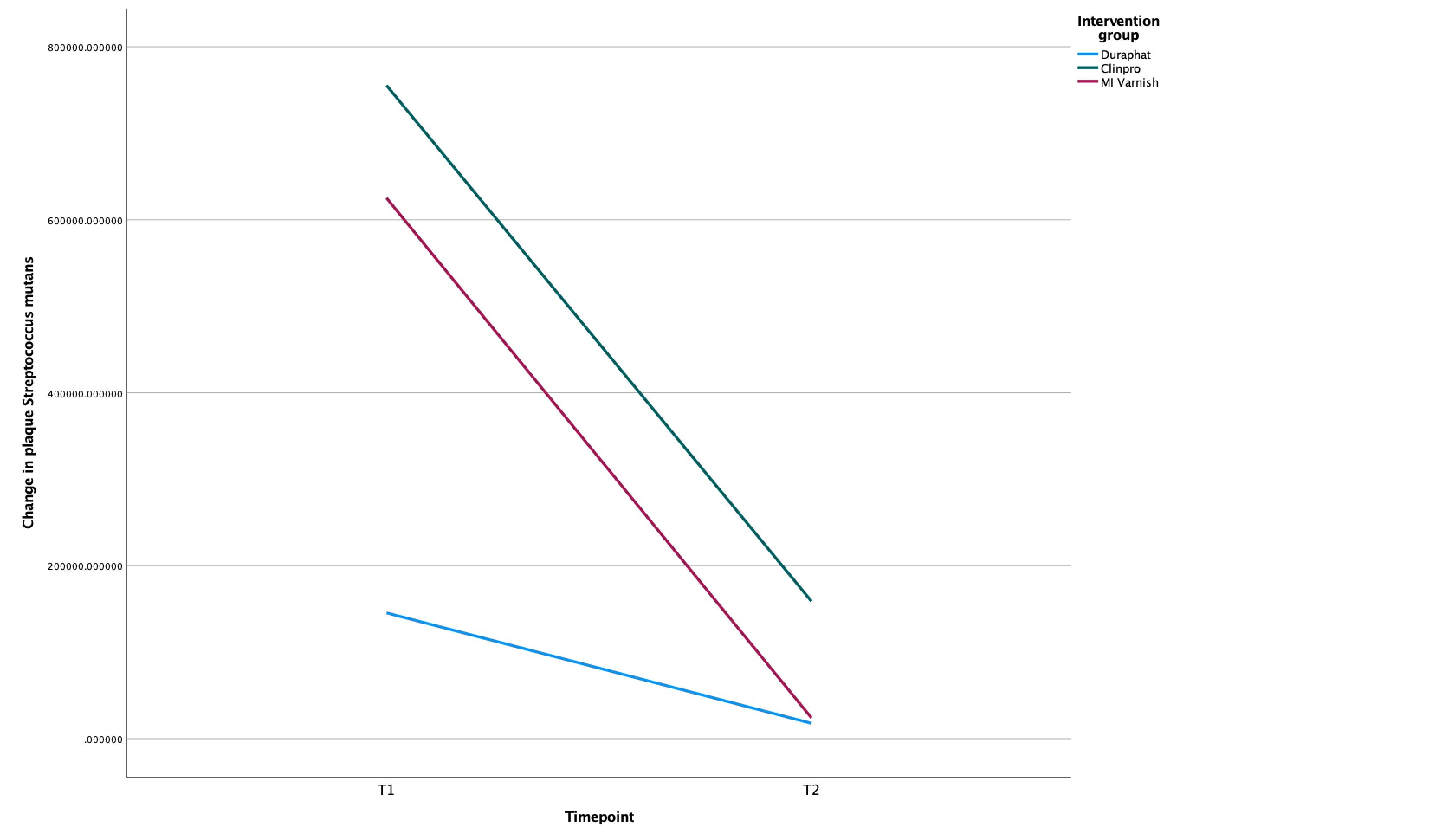
**

**Supplementary Figure 4: Mean change in salivary *L. fermentum* across intervention groups from baseline (T1) to 24-month final follow-up (T2)**

**
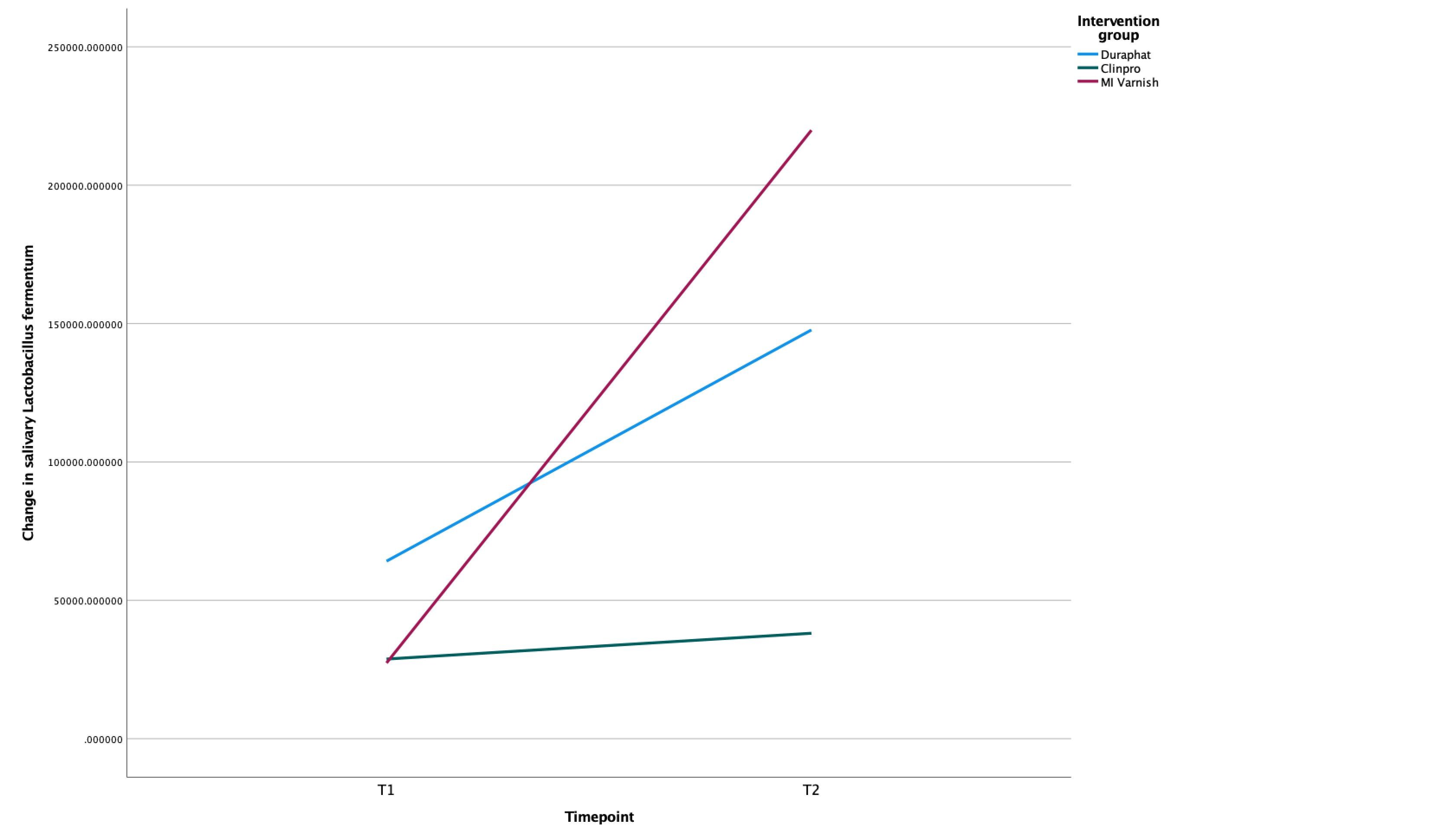
**

**Supplementary Figure 5: Mean change in plaque *L. fermentum* across intervention groups from baseline (T1) to 24-month final follow-up (T2)**

**
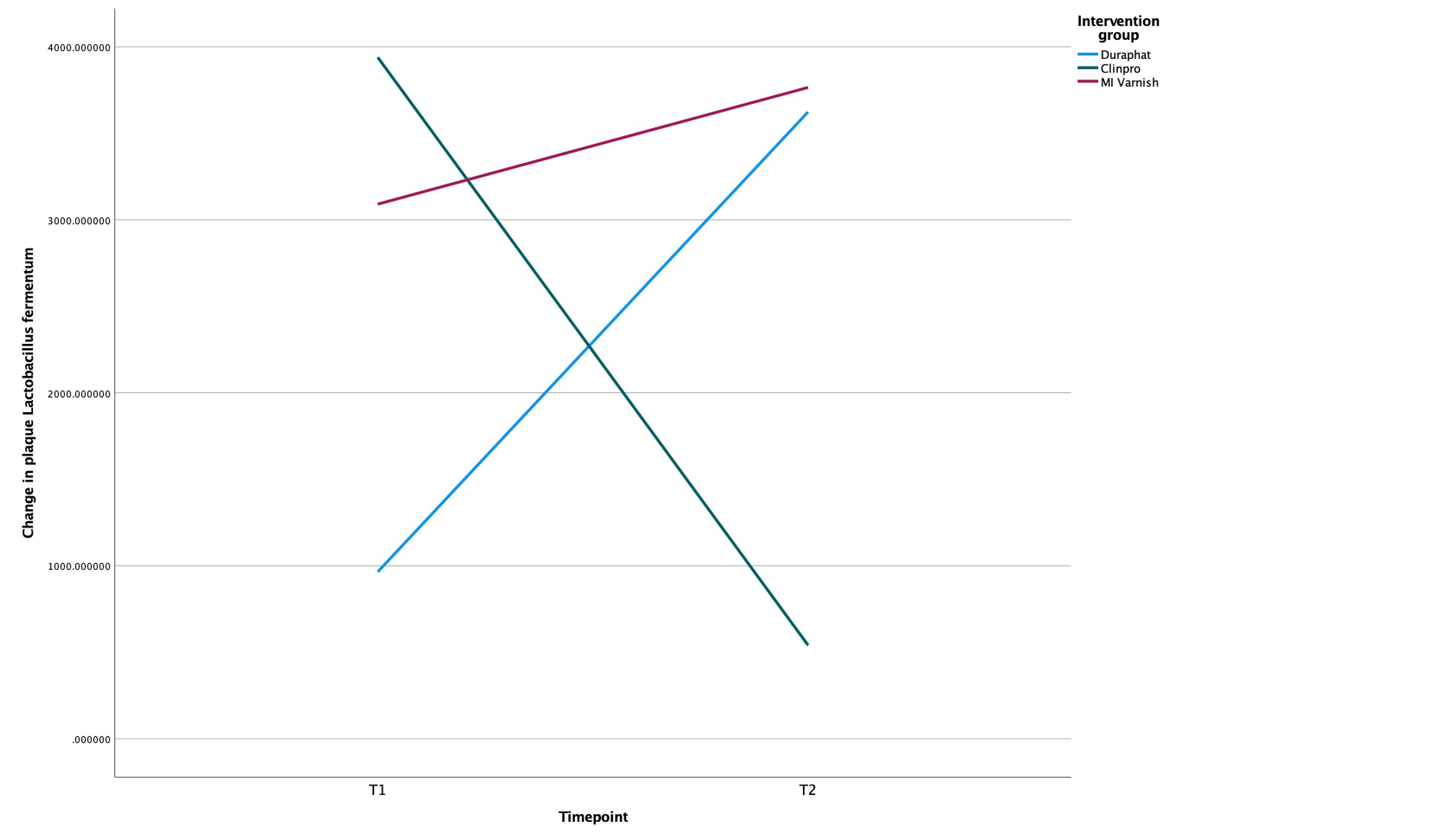
**

**Supplementary Figure 6: Mean change in salivary *S. mutans* across intervention groups according to the total number of fluoride applications**

**
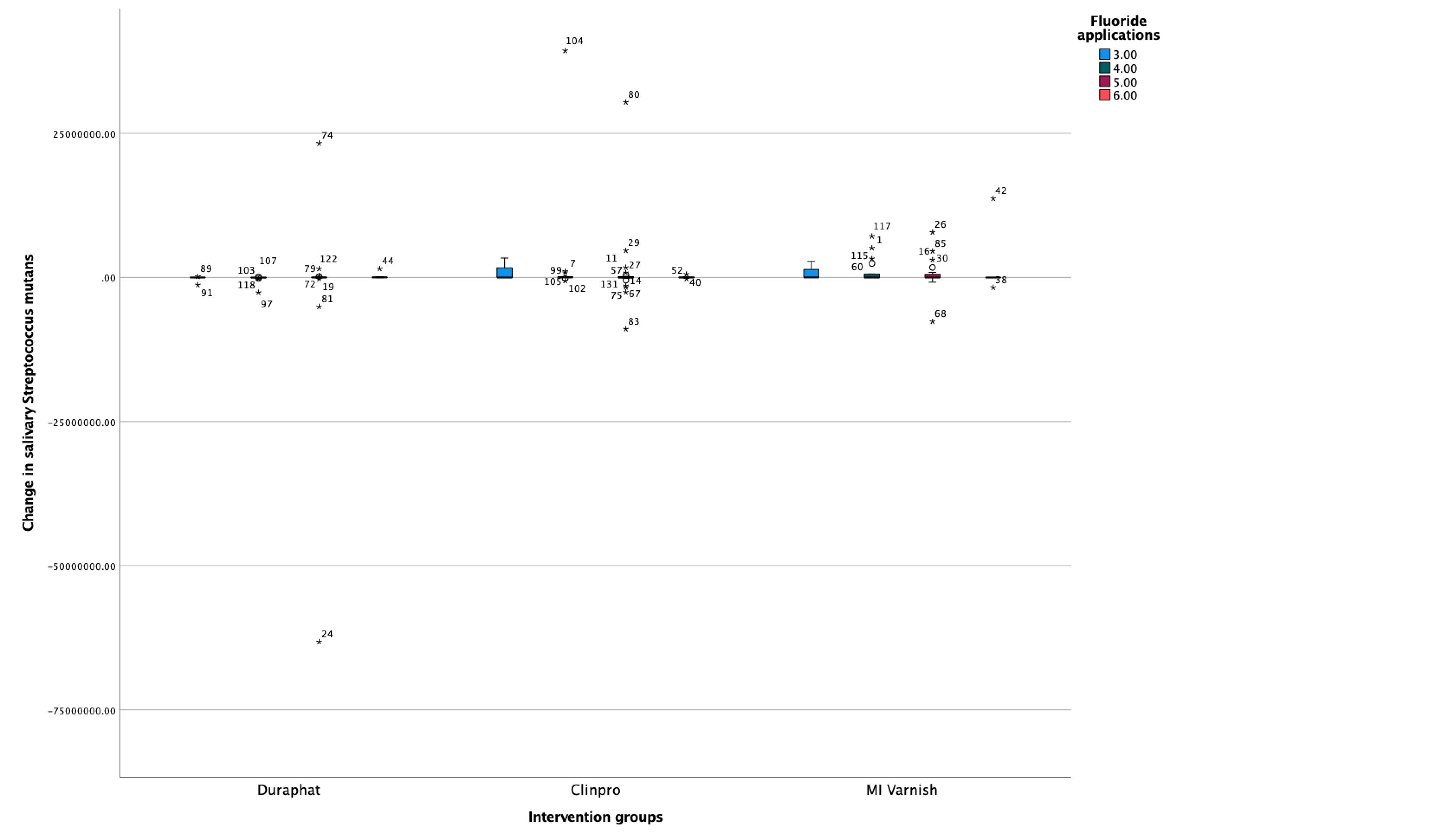
**

**Supplementary Figure 7: Mean change in plaque *S. mutans* across intervention groups according to the total number of fluoride applications**

**
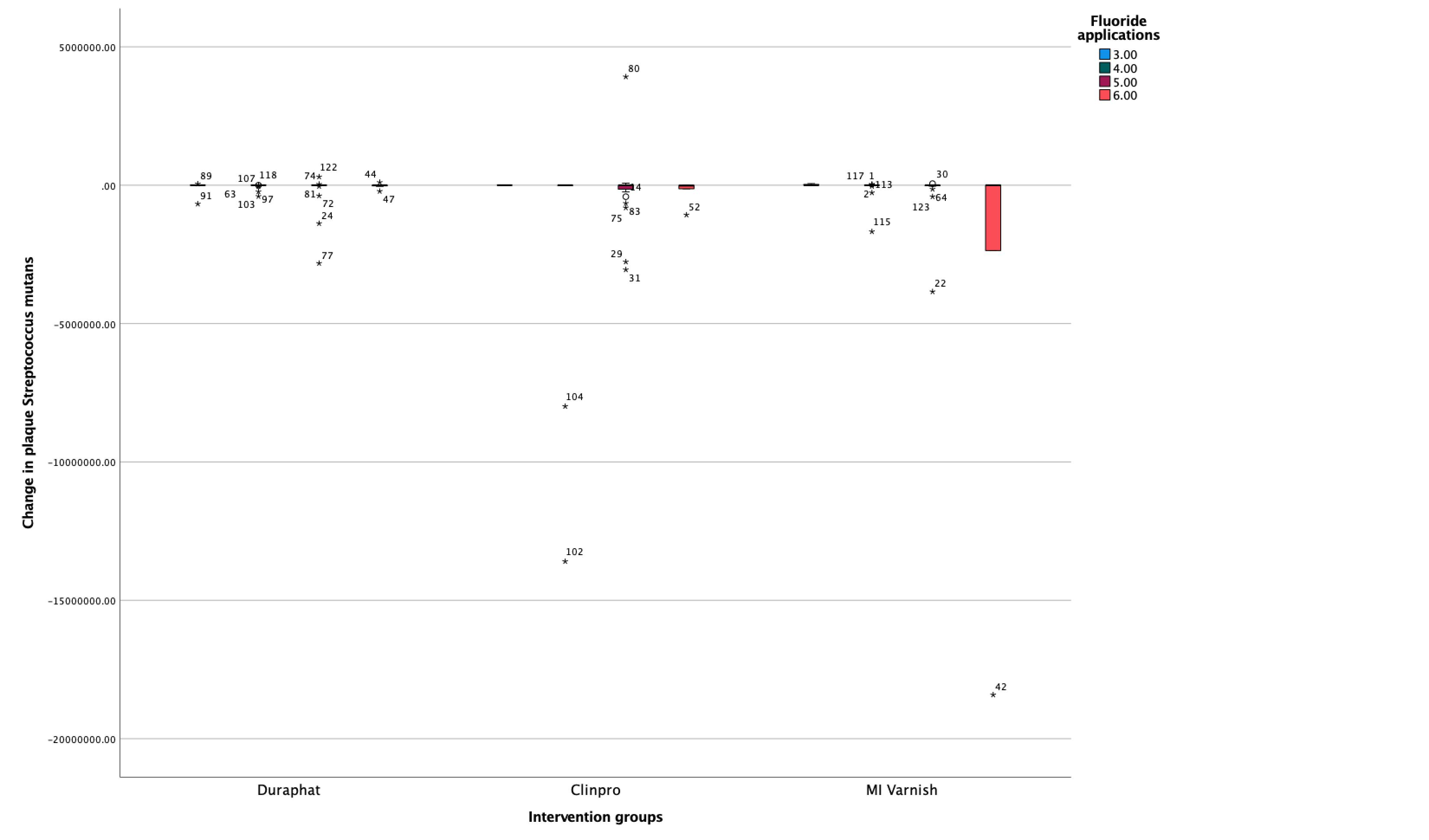
**

**Supplementary Figure 8: Mean change in salivary *L. fermentum* across intervention groups according to the total number of fluoride applications**

**
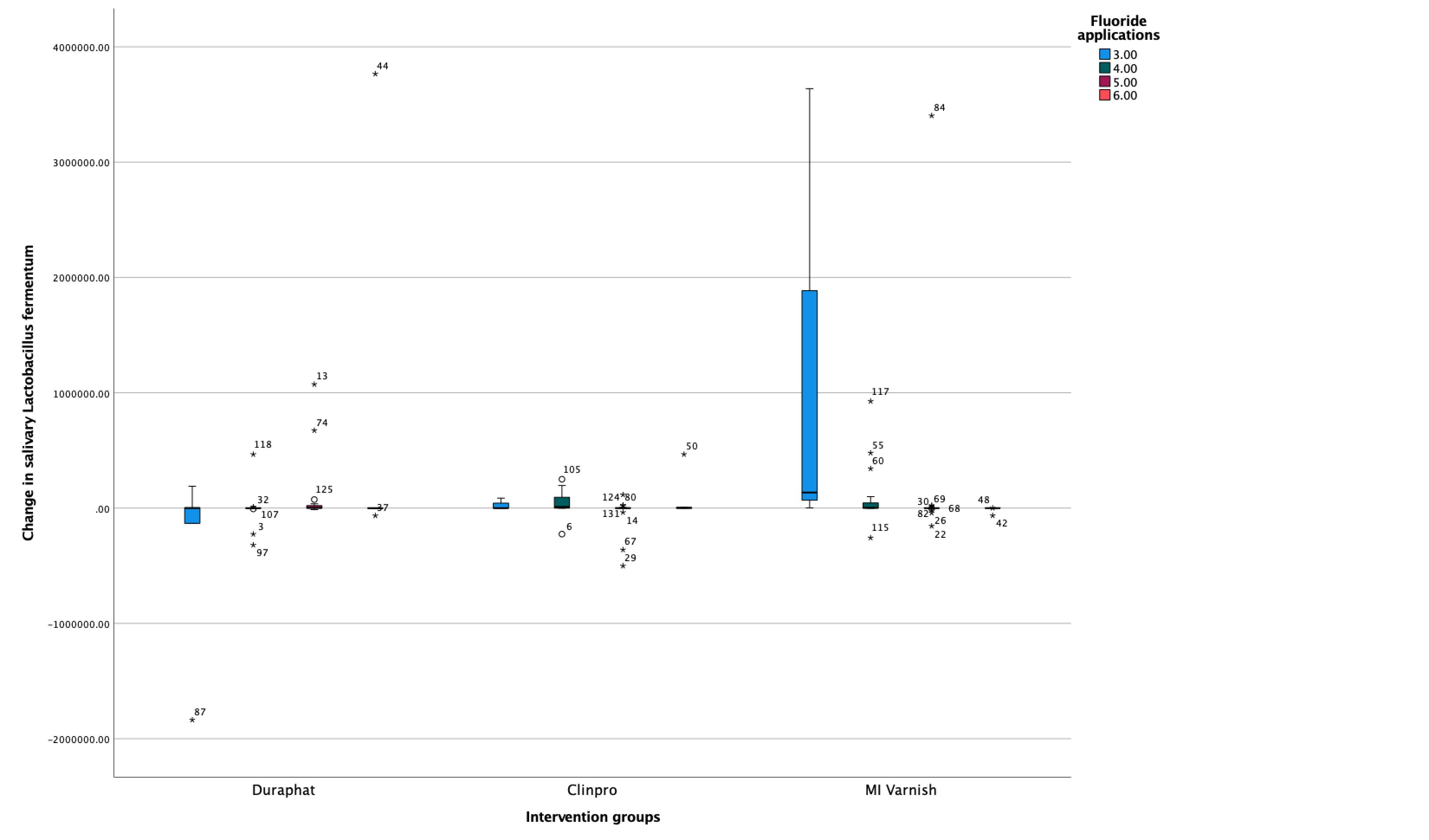
**

**Supplementary Figure 9: Mean change in plaque *L. fermentum* across intervention groups according to the total number of fluoride applications**

**
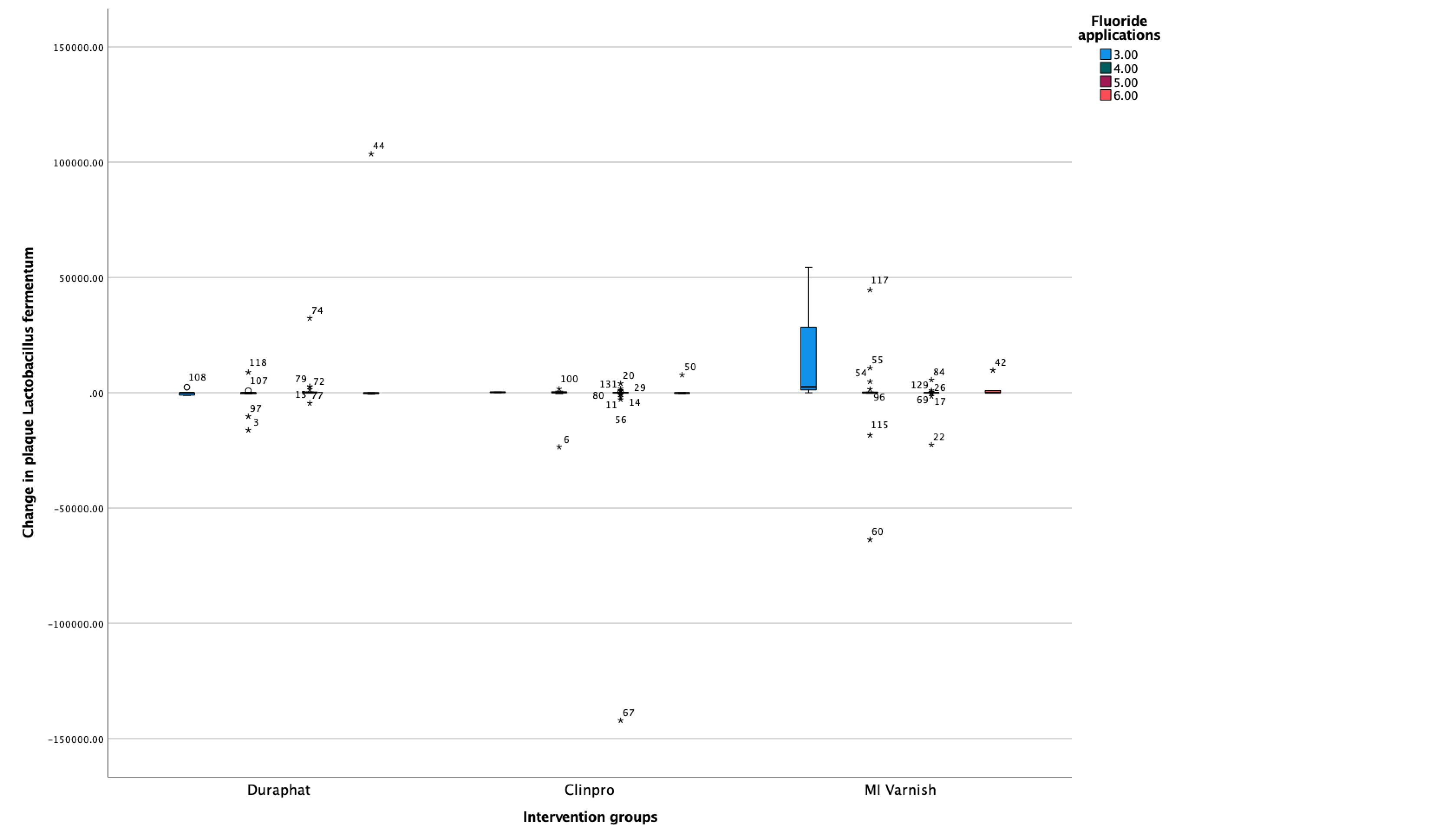
**
